# Physiological adaptation strategies for thermoregulation in *Tupaia belangeri* under high-temperature environment challenge

**DOI:** 10.3389/fphys.2025.1651991

**Published:** 2025-08-28

**Authors:** Dongjie Liu, Wanlong Zhu

**Affiliations:** ^1^ Key Laboratory of Ecological Adaptive Evolution and Conservation on Animals-Plants in Southwest Mountain Ecosystem of Yunnan Province Higher Institutes College, Yunnan Normal University, Kunming, China; ^2^ School of Life Sciences, Yunnan Normal University, Kunming, China; ^3^ Key Laboratory of Yunnan Province for Biomass Energy and Environment Biotechnology, Yunnan Normal University, Kunming, China

**Keywords:** Tupaiidae, thermoregulation, rest metabolic rate, thermal neutral zone, thermal conductance, tropical origin

## Abstract

**Introduction:**

To investigate the capacity of *Tupaia belangeri* to withstand high-temperature environments and its adaptability to global warming trends, while examining evidence for the species’ tropical origins through thermal neutral zone analysis.

**Methods:**

This study subjected *T. belangeri*, a representative mammal of the Oriental realm, to a temperature of 35 °C for 28 days to induce thermal acclimation. Body temperature (T_b_) and basal metabolic rate (BMR) were measured at ambient temperatures (T_a_) of 20 °C, 25 °C, 30 °C, 32.5 °C, 35 °C, and 37.5 °C, with thermal conductance (C) subsequently calculated. Latitudinal distributions and thermal neutral zone (TNZ) of 90 small mammals were compared against both normal-temperature and high-temperature acclimated *T. belangeri*.

**Results:**

Results indicated that T_b_ increased with rising ambient temperature, averaging 39.9 °C ± 0.16 °C within the TNZ. BMR showed no significant difference within the 30 °C–35 °C range. The mean BMR was 1.60 ± 0.025 mL O_2_/(g·h), indicating TNZ convergence at 30 °C–35 °C under high-temperature conditions. The mean C values within this range were 0.16 ± 0.0052mL O_2_/(g·h· °C). Compared to previous data on normal-temperature acclimation from our laboratory, high-temperature acclimated animals exhibited elevated T_b_, reduced BMR, a narrowed TNZ with an increased lower thermal neutral zone (LTNZ), and heightened C values. The TNZ of both acclimation groups in within the tropical high-temperature ranges.

**Discussion:**

These findings collectively indicated that *T. belangeri* adapts to thermal stress through increased T_b_, reduced metabolic rate, enhanced heat dissipation capacity, and a shift of the TNZ towards higher temperatures. Additionally, the TNZ of *T. belangeri* exhibited minimal fluctuations when subjected to high-temperature stress, indicating a strong adaptive capacity to warmer environments. Furthermore, the TNZ of *T. belangeri* is situated within the tropical high-temperature range, providing physiological evidence of its tropical origin based on the characteristics of the TNZ.

## 1 Introduction

Global warming has resulted in an increase in the frequency of high-temperature events, posing significant challenges to the survival and adaptability of several animal species. Research indicates an approximate 1.1 °C rise in mean global surface temperature over the past 50 years, with projections suggesting an additional increase of 1.5 °C–4.5 °C by the end of the century (Zhao et al., 2024). This rapid climatic shift compels small mammals to maintain energy balance and thermal stability through adjustments in thermoregulation, heat production capacity, or habitat selection (Chen et al., 2022). Environmental temperature profoundly influences both the physiological and morphological characteristics of small mammals, simultaneously regulating their energy management (Bi et al., 2018). In response to changes in environmental temperature, different taxa employ distinct adaptive strategies to cope with environmental fluctuations and ensure their survival (Hua et al., 2010). Notably, under conditions of high-temperature acclimation, *Cricetulus barabensis* increases its body mass (Zhang et al., 2025), while CD-1 mice exhibit a reduction in mass following thermal exposure (Bridges et al., 2012).

As a fundamental research area within physiological ecology, thermoregulation elucidates the critical relationship between environmental temperature and body temperature in small mammals. Research indicates that organisms exchange energy with their environment through several heat transfer mechanisms, including radiation, conduction, convection, and evaporation (Adam et al., 2017). For small mammals, the ability to maintain a consistently high body temperature is a key determinant of their survival range and geographical distribution (Zhu et al., 2008). For example, exposure to cold induces the proliferation of brown adipose tissue (BAT) in *Lasiopodomys brandtii*, enhancing thermogenic capacity through the activation of uncoupling protein (UCP), which increase energy expenditure during cold stress (Hou et al., 1999). Conversely, in response to thermal challenges, mammals typically maintain heat balance by reducing metabolic rates, enhancing heat dissipation efficiency, or employing behavioral adaptations. Desert rodents exemplify this strategy through nocturnal locomotion and reduced activity to minimize heat accumulation (Salaün et al., 2024). Additionally, *Meriones unguiculatus* significantly lowers its body temperature below control levels by actively suppressing metabolic heat production, thereby reducing both energy expenditure and water loss (Guo et al., 2020).

Basal metabolic rate (BMR) represents the energy expenditure required to sustain basic life functions in mammals and serves as a critical indicator of their capacity for environmental adaptation. Importantly, BMR levels are regulated by the thermal neutral zone (TNZ) (Feng et al., 2022), that is defined as the range of environmental temperatures within which mammals can maintain their body temperature without incurring additional energy costs. The width of the TNZ reflects the species’ ability to adapt to temperature. When environmental temperatures exceed the boundaries of the TNZ, mammals must engage in behavioral or physiological thermoregulation to maintain homeostasis (McAllister et al., 2009). Thermal conductance (C), a principal determinant of heat exchange between animals and their environment, is influenced by morphological factors such as body conformation, pelage density, and circulatory efficiency. These factors constitute a key component of energetic expenditure (Naya et al., 2013). For example, *Ochotona curzoniae*, which inhabits high-altitude environments, achieves enhanced thermal insulation through reduced C values (Zhu et al., 2022).

*Tupaia belangeri*, a small mammal endemic to the Oriental realm and belonging to the family Tupaiidae within the order Scandentia, primarily inhabits warm, humid broadleaf forests and mixed coniferous-broadleaf ecosystems. Its distribution encompasses Southeast Asia, India, Nepal, and Myanmar, with populations in China concentrated in Yunnan, southwestern Sichuan, southwestern Guizhou, southern Guangxi, and Hainan Island. Yunnan, Sichuan, and Guizhou represent the northern limits of this species’ distribution; it is hypothesized that this limitation is linked to its tropical origin and limited adaptability to low-temperature environments (Bremer et al., 2011). Previous research has demonstrated that *Tupaia belangeri*, which has expanded into the Hengduan Mountains, exhibits physiological adaptability by combining transitional traits of tropical animals with specific adaptations to the region. For example, low temperatures increase its metabolic rate; however, the extent of non-shivering thermogenesis enhancement diminishes with prolonged cold acclimation, which contrasts with the adaptations observed in northern small mammals (Zhang et al., 2012). Furthermore, its weight regulation differs from that of sympatric rodent species: under winter or cold conditions, it gains weight through a unique adaptive strategy (Feng et al., 2022). However, given the accelerating global warming and rising temperatures, a critical question arises: can *T. belangeri*, a species of tropical origin, adapt to thermal stress within its colonized habitat in the Hengduan Mountains? This adaptive capacity fundamentally depends on thermoregulatory competence and the efficacy of body mass regulation. Currently, no conclusive evidence exists regarding the precise mechanisms or extent of high-temperature adaptation in this species. Consequently, this study investigated *T. belangeri* specimens from Tuanjie Village, Kunming, analyzing changes in T_b_, BMR and C values under high-temperature acclimation conditions. Through a comparative assessment of TNZ dynamics, we examined the coping mechanisms and adaptive capacity of this species in high-temperature environments under global warming scenarios, while also verifying the possibility of its tropical origin from the perspective of TNZ. We predict that heat-stressed *T. belangeri* will exhibit: elevated T_b_, reduced BMR, a migration of TNZ towards higher temperature ranges, and increased C, which can provide evidence supporting the tropical origin of *T. belangeri* in terms of thermal neutrality.

## 2 Materials and methods

### 2.1 Animal collection

Experimental *T. belangeri* specimens were captured in Tuanjie Village, Kunming (average temperature: 14.9 °C; coordinates: 102°10′–102°40′E, 24°23′–25°03′N; terrain: north-high, south-low; altitude: 2,202 m) using rat cages. Following disinfection and defleaing, the animals were transported to the animal facility at Yunnan Normal University for individual housing under controlled conditions (temperature: 30 °C ± 1 °C; photoperiod: 12L: 12D). The subjects were provided *ad libitum* access to standardized feed (from Kunming Medical University) and water. All specimens were adults in a non-reproductive phase. Based on previous research indicating that the TNZ of *T. belangeri* spans 27.5 °C–35.0 °C; therefore, The acclimation temperature for high-temperature exposure in this study was set to 35 °C. Animals (*n* = 42, ♀:♂ = 18:24) underwent a 28-day acclimation period at 35 °C with a 12L:12D photoperiod. Post-acclimation measurements included T_b_, RMR, and calculated C values. All procedures complied with the regulations of the Medical Bioethics Committee of Yunnan Normal University (ethical approval: 13-0901-011).

### 2.2 Latitudinal coordinates and TNZ data sourcing for small mammals

The dataset for this study, which focuses on small mammals and *T. belangeri*, includes latitudinal coordinates and thermal neutral zones for species ranging from tropical to temperate environments, as detailed in Table 1. The dataset comprises a total of 91 species, spanning from *Gerbillus pusillus*, which has the lowest latitudinal coordinates, to *Sorex minutus*, which has the highest latitudinal coordinates.

**TABLE 1:** Latitudinal coordinates and thermal neutral zones of small mammals and *Tupaia belangeri* across different latitudinal regions.

Species	Latitude (°N)	LTNZ (°C)	UTNZ (°C)	TNZ (°C)	References
*Tupaia belangeri*	24.23	30	35	5	The present study
*Tupaia belangeri*	24.8	27.5	35	7.5	Zhang et al. (2012)
*Abrothrix longipilis*	−45.541	27.3	32	4.7	Bozinovic and Rosenmann (1989)
*Anoura latidens*	−18.293	34.7	36.2	1.5	Soriano et al. (2002)
*Apodemus agrarius*	48.29	25	27.5	2.5	Liu et al. (2004)
*Apodemus chevrieri*	26.15	20	27.5	7.5	Zhu et al. (2016)
*Apodemus speciosus*	48.923	25	30	5	Liu et al. (2004)
*Artibeus jamaicensis*	−20.054	25	35	10	McNab (1969)
*Auliscomys boliviensis*	−34.461	22.7	31	8.3	Bozinovic and Rosenmann (1989)
*Baiomys taylori*	28.01	29	36	7	Hudson (1965)
*Blarina brevicauda*	42.273	25	33	8	Deavers and Hudson (1981); Neal and Lustik (1973)
*Brachylagus idahoensis*	41.6	17.5	25.5	8	Katzner and Parker (1997)
*Cannomys badius*	15.52	26.74	34.5	7.76	McNab (1979)
*Carollia perspicillata*	−20.056	29	35	6	McNab (1969)
*Cercartetus concinnus*	−30	28	30	2	Geiser (1987)
*Cryptomys damarensis*	−25.47	27	31	4	Lovegrove (1986)
*Ctenomys talarum*	−37.46	25	30	5	Busch et al. (1989)
*Cynopterus brachyotis*	4.53	30	37	7	McNab (1989)
*Desmodus rotundus*	−20.053	29	37.5	8.5	McNab (1969)
*Diaemus youngi*	−41.013	25	30	5	McNab (1969)
*Didelphis marsupialis*	23.38	25.5	36	10.5	McNab (1978)
*Dipodomys microps*	36.052	27	32	5	Breyen et al. (1973)
*Dobsonia minor*	−5.28	27.5	35	7.5	Bartholomew et al. (1970)
*Dolichotis salinicola*	−23.26	28	37	9	Arends and McNab (2001)
*Eothenomys miletus*	25.03	22.5	30	7.5	Zhu et al. (2008)
*Eothenpmys olitor*	27.3	20	27.5	7.5	Yang et al. (2021)
*Erinaceus concolor*	50	27.5	31.5	4	Krol (1994)
*Geomys pinetis*	29.4	26	35	9	McNab (1966)
*Gerbillurus paeba*	−23.29	32.3	35.1	2.8	Buffenstein (1984)
*Gerbillurus setzeri*	−24.08	32.2	34.8	2.6	Dempster et al. (1998)
*Gerbillurus tytonis*	−29.04	32.4	34.9	2.5	Downs and Perrin (1990)
*Gerbillurus vallinus*	−22.52	33.1	35	2.9	Dempster et al. (1999)
*Gerbillus pusillus*	0.38	31.4	38	6.6	Buffenstein and Jarvis (1985)
*Glossophaga longirostris*	11.472	31.5	36	4.5	Arends et al. (1995)
*Glossophaga soricina*	−6.11	31.4	35.2	3.8	Cruz-Neto and Abe (1997)
*Heterocephalus glaber*	−2.45	31	37	6	McNab (1966)
*Kerodon rupestris*	−12.31	27	36	9	Arends and McNab (2001)
*Loxodontomys micropus*	−34.57	22	33	11	Bozinovic and Rosenmann (1989)
*Macroderma gigas*	−23.299	30	35	5	Baudinette et al. (2000), Leitner and Nelson (1967)
*Macrotis lagotis*	−26.08	27	35	8	Kinnear and Shield (1975)
*Megadontomys thomasi*	17.55	28	35	7	Buffenstein and Jarvis (1985)
*Metachirus nudicaudatus*	9.33	27.5	36	8.5	McNab (1978)
*Microcebus murinus*	48.7	25	28	3	Aujard et al. (1998)
*Microtus arvalis*	52.34	20	30	10	Jansky (1959)
*Microtus montanus*	46.43	26	31	5	Packard (1968)
*Microtus pennsylvanicus*	45.54	25	29	4	Wiegert (1961)
*Miniopterus schreibersii*	−12.275	32.5	37.5	5	Baudinette et al. (2000)
*Molossus molossus*	−20.492	32	36	4	McNab (1969)
*Monodelphis brevicaudata*	10.42	28.76	36	7.24	McNab (1978)
*Monophyllus redmani*	18.391	31	35	4	Rodriguez-Duran (1995)
*Mormoops blainvillei*	18.397	30	36	6	Rodriguez-Duran (1995)
*Mormoops megalophylla*	11.48	33.5	39.5	6	Bonaccorso et al. (1992)
*Natalus tumidirostris*	11.471	28	35	7	Genoud et al. (1990)
*Neomys anomalus*	52.77	25	30	5	Gebczynska and Gebczynski (1965)
*Neomys fodiens*	52.9	25	30	5	Gebczynska and Gebczynski (1965)
*Neurotrichus gibbsii*	49.3	25	32	7	Campbell and Hochachka (2000)
*Noctilio albiventris*	−20.055	32	38	6	McNab (1969)
*Noctilio leporinus*	−20.051	28	38	10	McNab (1969)
*Notomys alexis*	−23.45	32	34	2	MacMillen and Lee (1969)
*Notomys cervinus*	−25.34	33	34	1	MacMillen and Lee (1969)
*Nycticebus pygmaeus*	23.07	27.5	35	7.5	Xiao C et al. (2010)
*Ochrotomys nuttalli*	29.15	29.5	36	6.5	Layne and Dolan (1975)
*Otomys irroratus*	−25.433	24	28	4	Haim and Fairall (1987)
*Peromyscus gossypinus*	29.397	29.7	35.3	5.6	Layne and Dolan (1975)
*Peropteryx macrotis*	11.25	30.5	37	6.5	Genoud et al. (1990)
*Petauroides volans*	−31.53	18	25	7	Rübsamen et al. (1984)
*Philander opossum*	9.01	29.5	36	6.5	McNab (1978)
*Phyllostomus discolor*	−20.491	25	37	12	McNab (1969)
*Planigale maculata*	−12.28	31	35	4	Morton and Lee (1978)
*Potorous tridactylus*	−42.541	20	30	10	Nicol (1976)
*Pteronotus davyi*	11.482	34.5	43	8.5	Bonaccorso et al. (1992)
*Pteronotus parnellii*	11.485	34	40.5	6.5	Bonaccorso et al. (1992)
*Pteronotus personatus*	11.488	34	38.5	4.5	Bonaccorso et al. (1992)
*Pteronotus quadridens*	1.914	30	38	8	Rodriguez-Duran (1995)
*Pteropus rodricensis*	−27.145	24	35.5	11.5	McNab and Bonaccorso (2001)
*Pteropus scapulatus*	−28.316	24	35	11	Bartholomew et al. (1964)
*Rattus villosissimus*	−22.59	30	35	5	Collins and Bradshaw (1973)
*Saccopteryx bilineata*	10.25	30	35	5	Genoud and Bonaccorso (1986)
*Sminthopsis crassicaudata*	−22.75	31	38	7	Geiser and Baudinette (1987)
*Sorex cinereus*	−37.79	25	30	5	Morrison et al. (1959)
*Sorex minutus*	53.28	24	30	6	McDevitt and Andrews (1994)
*Spermophilus beecheyi*	33.412	25	30	5	Baudinette (1972)
*Sturnira lilium*	−20.052	30	35.5	5.5	McNab (1969)
*Tachyoryctes splendens*	−1.77	27	35	8	McNab (1966)
*Tamiasciurus hudsonicus*	50.29	15	27	12	Pauls (1981)
*Tarsius syrichta*	9.849	32	35	3	McNab and Wright (1987)
*Thallomys paedulcus*	−25.1	27.46	35.89	8.43	Lovegrove et al. (1991)
*Thomomys talpoides*	37.44	26	32	6	Bradley et al. (1974)
*Thylamys elegans*	−23.445	27.5	35	7.5	Bozinovic et al. (2005)
*Tupaia glis*	10.32	30	37	7	Bradley and Hudson (1974)
*Vulpes zerda*	33.418	23.4	32	8.6	Noll-Banholzer (1979)

### 2.3 Determination of T_b_, BMR and C values

Body mass, T_b_, and BMR were measured in seven specimens across ambient temperatures of 20 °C, 25 °C, 30 °C, 32.5 °C, 35 °C, and 37.5 °C. Mass determination was conducted using an analytical balance (AB204-S, Mettler Toledo, Switzerland; accuracy ±0.01 g). Body temperature was recorded after each metabolic rate test at each temperature. Rectal temperature of animals was measured, using a digital thermometer (XGN-1000T, Beijing Yezhiheng Technology Co. Ltd.; accuracy ±0.1 °C). The probe of the thermometer was inserted 3 cm into the rectum and a reading was taken after 30 s. BMR measurements utilized a small-mammal metabolic system (PRO-MRMR-8, Able Systems International Inc.). Following a fasting period of 2–4 h, the animals were placed in metabolic chambers with an airflow rate of 2 L/min under thermoneutral conditions. After more than 30 min of acclimation to a resting state, data were recorded at 5-min intervals for a duration of 60 min (Zhu et al., 2008). Post-experiment, two consecutive stable minimum values were selected for RMR calculation. The C value was derived using McNab’s equation: C = BMR/(T_b_ - T_a_) (McNab, 2009), where T_b_ denotes body temperature and T_a_ denotes ambient temperature.

### 2.4 Statistical analysis

Data were analyzed using the SPSS v26.0 software package. Prior to all statistical analyses, the data were tested for normality and homogeneity of variance using the Kolmogorov-Smirnov and Levene tests, respectively. Differences between sexes for the physiological indices of *T. belangeri* were not significant; therefore, all data were combined and analyzed collectively. T_b_ was assessed using a one-way ANOVA, while RMR and C values were analyzed using ANCOVA, with body mass as a covariate. A linear regression was employed to model the correlation between TNZ and latitudinal coordinates, as well as the relationships between T_a_ and T_b_, RMR, and C values. Results was expressed as mean ± standard error (SE), with statistical significance set at *p* < 0.05.

## 3 Results

### 3.1 Body temperature

**FIGURE 1:**
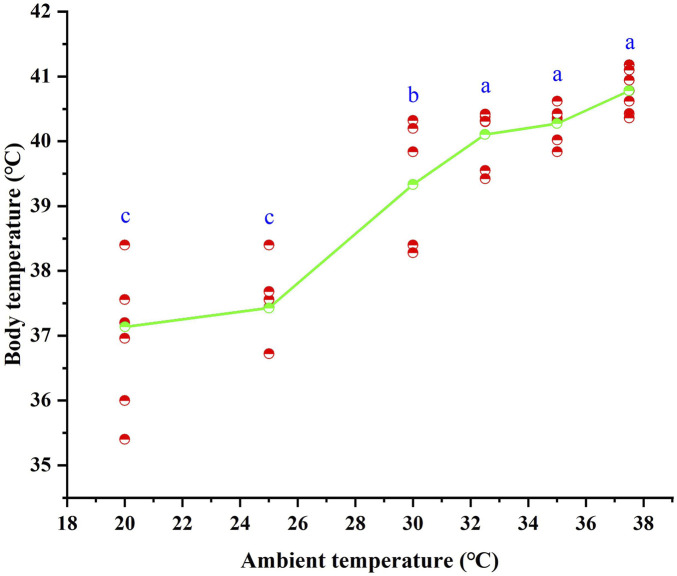
Body temperature of *Tupaia belangeri* at varying ambient temperatures. Different letters (a, b and c) indicate statistically significant differences in body temperature data between groups (*p* < 0.05), and the same letter represents no significant difference; the scatter points are individual measured values, and the green line show the mean fitted trend.

Following 28 days of high-temperature acclimation, the T_b_ of *T. belangeri* ranged from 35.4 °C to 41.18 °C. An ANOVA revealed significant positive correlation between T_b_ of *T. belangeri* and the ambient temperature ranging from 20 °C to 37.5 °C. T_b_ increased with rising ambient temperature, and the linear regression equation relating T_b_ to T_a_ was expressed as T_b_ = 32.259 + 0.23*T_a_ (*R* ^*2*^ = 0.789, *F* = 149.852, *p* < 0.01), (Figure 1).

### 3.2 Resting metabolic rate (RMR) and TNZ

**FIGURE 2:**
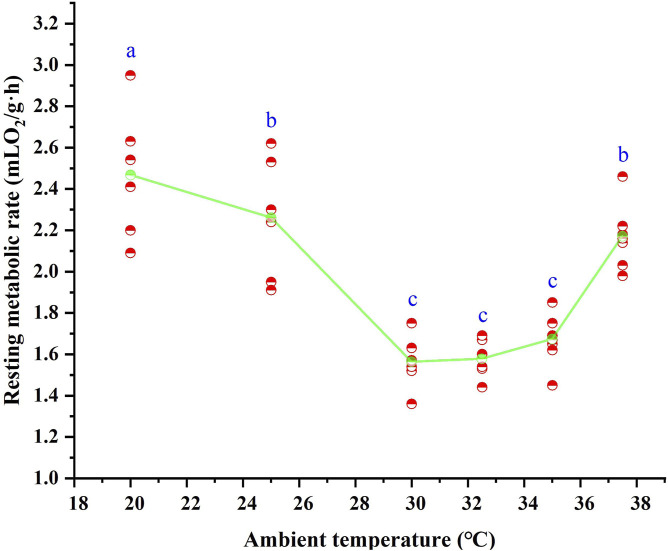
Resting metabolic rate of *Tupaia belangeri* at varying ambient temperatures. Different letters (a, b and c) indicate statistically significant differences in resting metabolic rate data between groups (*p* < 0.05), and the same letter represents no significant difference; the scatter points are individual measured values, and the green line show the mean fitted trend.

Following 28 days of high-temperature acclimation, the RMR of *T. belangeri* showed a highly significant variation in response to ambient temperature (*F* = 31.848, *p* < 0.01). RMR increased as ambient temperature decreased below 30 °C (Figure 2), revealing a linear relationship with temperature. The regression equation for RMR as a function of ambient temperature in the 20 °C–30 °C range was RMR = 4.349–0.09*T_a_ (*r* ^2^ = 0.692, F = 42.673, *p* < 0.01). Above 35 °C, RMR also increased with rising temperature (Figure 2), with a linear regression described by RMR = −5.291 + 0.199*T_a_ (*r* ^2^ = 0.789, F = 44.974, *p* < 0.01) across the 35 °C–35.7 °C range. ANOVA revealed that RMR remained stable between 30 °C and 35 °C (F = 2.058, *p* > 0.05), representing the BMR with a mean of 1.60 ± 0.025 mL O_2_/(g·h). This value differed significantly from RMR at 25 °C and 37.5 °C (*p* < 0.05), defining the thermal neutral zone (TNZ) as 30 °C–35 °C. The mean body temperature within the TNZ was 39.9 °C ± 0.16 °C.

### 3.3 C values

**FIGURE 3:**
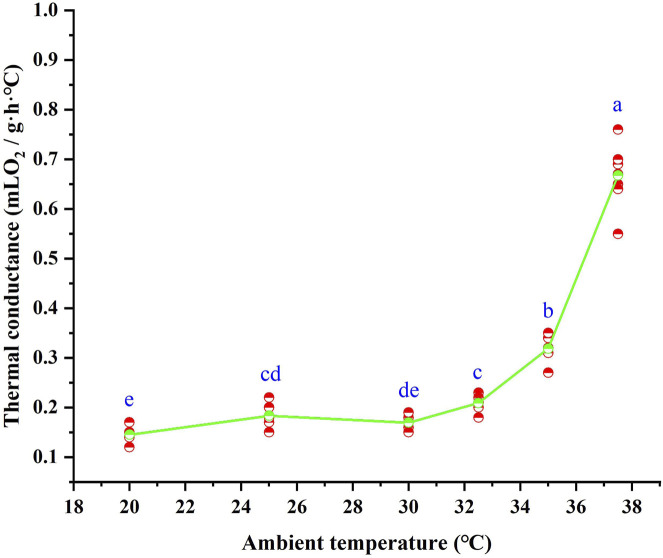
Thermal conductance of *Tupaia belangeri* at varying ambient temperatures. Different letters (a, b, c, d and e) indicate statistically significant differences in thermal conductance data between groups (*p* < 0.05), and the same letter represents no significant difference; the scatter points are individual measured values, and the green line show the mean fitted trend.

Following 28 days of high-temperature acclimation, the C values of *T. belangeri* varied significantly with ambient temperature (*F* = 279.642, *p* < 0.01). The C values were lowest below the LTNZ, where they remained relatively stable. Within the TNZ, C values increased with rising ambient temperature, averaging 0.16 ± 0.0052 mL O_2_/(g·h· °C). This relationship was described by the linear regression equation C = −0.740 + 0.03*T_a_ (*R* ^*2*^ = 0.866, *F* = 122.403, *p* < 0.01). Beyond the upper thermal neutral zone (UTNZ), C values increased sharply with further elevation in ambient temperature (Figure 3).

### 3.4 Relationship between TNZ and latitude coordinates

**FIGURE 4:**
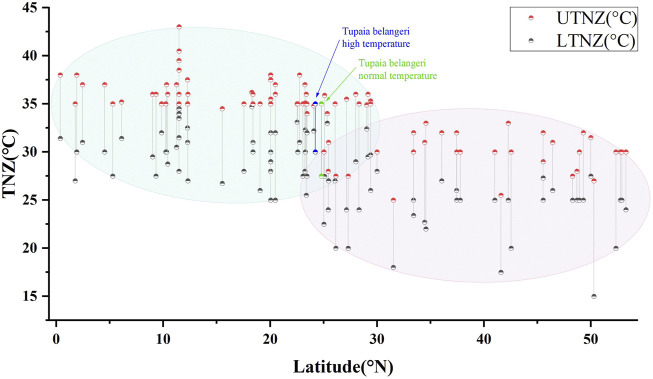
Relationship between the TNZ and latitudinal coordinates in *Tupaia belangeri* and 90 small mammal species.

Comparative analysis of thermal neutral zones for the 91 small mammal species along latitudinal gradients, including TNZ data for *T. belangeri* under normothermic and high temperature acclimation conditions, revealed significant correlations between latitudinal distribution and TNZ boundaries. The LTNZ of small mammals exhibited a strong correlation with latitude (*R* ^*2*^ = 0.365, *F* = 51.78, *p* < 0.01), as did the UTNZ (*R* ^*2*^ = 0.537, *F* = 104.398, *p* < 0.01). Collectively, these findings show that small mammal thermal neutral zones are significantly dependent on latitude, with low-latitude species displaying higher TNZ ranges than their high-latitude counterparts (Figure 4).

## 4 Discussion

Faced with environmental changes, animals maintain energy homeostasis by modulating physiological and ecological traits (Stawski and Simmonds, 2021). Body temperature is central to energy regulation and serves as a key physiological marker in animal energetics research (Ayres, 2020). For instance, rats adapted to extreme cold actively reduce their body temperature to minimize heat production at −30 °C (Yin et al., 2009), while *Phodopus roborovskii* elevates its body temperature through increased non-shivering thermogenesis in cold environments (Bao et al., 2001). Previous studies have shown that *T. belangeri* regulates energy by lowering its body temperature under cold stress (Zhang et al., 2001). In our study, the body temperature of *T. belangeri* increased with ambient temperature (20 °C–37.5 °C) following high-temperature acclimation (Figure 4), consistent with findings in *Tupaia glis* (Bradly et al., 1974) and *Crocidura suaveolens* (Wang et al., 2010). This would that species within the family Tupaiidae exhibit common physiological characteristics and unique body temperature regulation patterns in response to varying ambient temperatures. Additionally, the body temperature of *T. belangeri* was lower at normothermia (39.7 °C) compared to after high-temperature acclimation (39.9 °C) (Table 2). This pattern is supported by findings in blackline hamsters (Zhao et al., 2018) and long-clawed gerbils (Guo et al., 2020), where thermal acclimation leads to an elevation in body temperature. Interestingly, this contrasts with the response of desert rodents like *Gerbillus pusillus*, which maintain lower body temperatures to minimize water loss (Buffenstein and Jarvis, 1985). These findings corroborate the results of our study and suggest that such physiological adjustments may represent an adaptive mechanism for high-temperature environments. By moderately increasing body temperature, animals can reduce their resting metabolic rate while enhancing thermal conductance, thereby decreasing the energy expenditure required to maintain a constant internal temperature and optimizing energy allocation. Furthermore, a suitable rise in body temperature can minimize the thermal gradient between the body and the external environment, effectively promoting heat dissipation and reducing heat production (Vejmělka et al., 2021). This strategy facilitates a balance between heat production and dissipation, thus preventing metabolic overload under high-temperature stress. Our results indicate that *T. belangeri* enhances heat dissipation by elevating body temperature to maintain thermal equilibrium. This adaptive response may represent an evolutionary strategy for coping with prolonged exposure to high-temperature conditions.

**TABLE 2:** Physiological indices of *Tupaia belangeri* under normal temperature and high-temperature acclimation.

Acclimation	Normal temperature 30 °C	High temperature 35 °C
Body temperature (°C)	39.7	39.9
Rest metabolic rate (mL O_2_/(g·h))	1.66	1.60
Thermal neutral zone (°C)	27.5–35	30.0–35
Thermal conductance (mL O_2_/(g·h·°C))	0.15	0.16
References	Huang et al. (2013)	The present study

RMR serves as a crucial indicator of energy expenditure in animals, influenced by several factors including temperature, food availability, and activity levels (such as exercise, reproductive behaviors, and stress responses) (Terblanche et al., 2007; Rising et al., 2015). Importantly, RMR plays a pivotal role in processes of environmental adaptation (Feierabend and Kielland, 2015). Research shows that cold-exposed *C. barabensis* exhibit elevated RMR and non-shivering thermogenesis compared to controls maintained at normal temperatures (Chen et al., 2014). Similarly, high-altitude *Lberomys cabrerae* maintain stable body temperatures through increased RMR (Castellanos-Frias et al., 2015), while cold-acclimated *P. roborovskii* show enhanced RMR and non-shivering thermogenesis capacity (Chi and Wang, 2011). Our findings indicate that *T. belangeri*, when exposed to thermal acclimation, exhibited a reduced RMR compared to laboratory measurements taken at normal temperatures. This pattern mirrors those observed in high-temperature-acclimated rodents from the Hengduan Mountains region, that include to *Eothenomys miletus*, *Eothenpmys olitor*, and *Apodemus chevrieri* (Geng and Zhu, 2024), suggesting a convergent evolutionary adaptation. In contrast, temperate rodents like *Microtus arvalis* often increase RMR under thermal stress to sustain thermogenesis (Jansky, 1959). The observed reduction in RMR likely represents an adaptive response to thermal stress, serving as a crucial strategy for minimizing heat production in warmer environments (Speers-Roesch et al., 2018). For *T. belangeri*, lower metabolic rates would help to prevent overheating while facilitating energy reallocation to essential functions such as foraging and reproduction. Additionally, reduced energy expenditure allows animals to maintain lower basal metabolic costs, thereby decreasing the time required for foraging. This adaptive strategy provides dual survival benefits by simultaneously reducing the risk of predation and offering a survival advantage during periods of food scarcity (Mónus and Barta, 2016; Shiratsuru et al., 2021). *Tupaia belangeri* exhibited an efficient energy adaptation strategy in response to warmer environmental by lowering its energy consumption. This approach reflects a highly effective mechanism for coping with climate change.

TNZ is a specific range of ambient temperatures within which animals maintain thermoregulation by regulating heat loss, without relying on metabolic heat production or evaporative heat dissipation (Kingma et al., 2014). Although multiple factors influence the TNZ, environmental temperature is a critical determinant (Yang et al., 2021). For the same species, the TNZ shifts with changes in environmental temperature: LTNZ decreases and the width of the TNZ increases in cold environments, whereas the LTNZ rises and the TNZ narrows in warm environments (Zhu et al., 2016). For instance, the species *Dipus sagitta* exhibits a narrow TNZ around 30 °C in spring, the widest TNZ and highest heat resistance in summer, and a gradual shift to lower temperatures in autumn (Bao et al., 2000). Across species, the TNZ correlates with altitude, temperature, and other environmental factors (Zhu et al., 2022). Our study shows that *T. belangeri* has a TNZ of 30 °C–35 °C under moderate to high temperatures. Previous laboratory research on *T. belangeri* indicates that high-temperature acclimation narrows the TNZ by elevating the lower critical temperature while maintaining the upper critical temperature. The reduction in TNZ during heat exposure is partly attributed to a decreased metabolic rate (Zhao et al., 2010; Scholander et al., 1950). The upward shift in the critical temperature of the TNZ suggests that thermal acclimation enhances *T. belangeri*’s temperature tolerance to higher ranges, thereby reducing energy costs for thermoregulation in warmer environments, improving heat resistance, and increasing survival rates. For small mammals, adjusting the TNZ is a crucial strategy for adapting to climate change (Zhao et al., 2018).

By integrating findings from relevant high-temperature acclimation researches conducted in our laboratory on other five small mammal species, we observed that high-temperature acclimation resulted in a narrowing of the TNZ and an increase in the LTNZ across all five species (Table 3). This suggests that high-temperature exposure causes the TNZ to become more specialized in these animals. In comparison, we found that the change in TNZ width for *T. belangeri* was smaller than that of *C*. *barabensis*, indicating that *T. belangeri* exhibits greater adaptability to high temperatures than northern species. Furthermore, the temperature range of critical temperature drift within the TNZ of *T. belangeri* is limited, which further suggests that it possesses strong thermal adaptation and does not require significant adjustments to its physiological functions in response to thermal changes.

**TABLE 3:** Comparison of the thermal neutral zone in related species under normal temperature and high-temperature acclimation.

Species	Normal temperature (°C)	High temperature (°C)	References
*Cricetulus barabensis*	25–32.5	30–32.5	Zhao et al. (2018)
*Eothenomys miletus*	22.5–30	25–30	Zhu et al, (2008); Geng and Zhu (2024)
*Eothenpmys olitor*	20–27.5	25–30	Yang et al. (2021); Geng and Zhu (2024)
*Apodemus chevrieri*	20–27.5	25–30	Zhu et al. (2016); Geng and Zhu (2024)
*Tupaia belangeri*	27.5–35	30–35	Huang et al. (2013); The present study

The origin and distribution patterns of *T. belangeri* remain subjects of ongoing debate; however, existing evidence supports a tropical-to-temperate dispersal trajectory (Roberts et al., 2011). Previous laboratory researches utilizing adaptive thermogenesis measurements and molecular ecological analyses have consistently indicated a south-to-north expansion pattern for this species, providing compelling support for its tropical origins (Fu et al., 2018; Jia et al., 2008; Zhang et al., 2009; Zhu et al., 2014). Our study compares the latitudes of regions inhabited by 90 small mammal species with the TNZ of *T. belangeri* under both normal and high-temperature acclimation. The findings reveal a correlation between TNZ and habitat latitude: high-latitude small mammals exhibit thermal neutral zones at higher temperature ranges than their low-latitude counterparts. Additionally, both the normal and high-temperature acclimated TNZ of *T. belangeri* is with the tropical high-temperature zones (Figure 2). The TNZ response to heat exposure reflects the species’ adaptive potential to extreme temperatures, which is crucial for determining whether it originated from tropical environments exposed to prolonged high-temperature stress (Wang et al., 2021). The TNZ of *T*. *belangeri* under heat exposure is still located in the tropical high temperature zone and the UTNZ remains unchanged, a characteristic feature of tropical species. This suggests that *T. belangeri* likely migrated from tropical areas to its current distribution, specifically from south to north, and may have developed adaptive physiological mechanisms to the high-temperature tropical environment over an extended evolutionary history. Its effective acclimation to high temperatures results as robust evidence for the likely tropical origin of *T. belangeri*.

Thermal conductance plays a pivotal role in the energy balance of small mammals, serving as one of the most critical factors influencing their energy expenditure (Naya et al., 2013). Environmental temperature profoundly affects thermal conductance: it decreases at low temperatures and increases at high temperatures, allowing animals to dissipate excess heat and maintain thermal stability along with a constant body temperature (Schmidt-Nielsen, 1997). For instance, *Phodopus sungorus* captured at low altitudes exhibit higher thermal conductance (Geiser et al., 2016). Small mammals struggle to adapt to fluctuating environmental temperatures by altering fur thickness, making adjustments in thermal conductance values essential (Yang et al., 2021). In our study, the C values remained relatively stable below the TNZ, while values above 30 °C increased with rising ambient temperatures. This thermal strategy reflects two possible adaptations: at lower temperatures, *T. belangeri* shows effective thermal insulation and heat dissipation blocking, which allows it to minimize energy expenditure for thermoregulation. However, in high-temperature environments, the reduced temperature difference between the body surface and the environment results in weaker heat dissipation capacity, preventing the timely release of metabolic heat and resulting in elevated body temperatures. This mechanism would partly explain why *T. belangeri*’s body temperature increases with ambient temperature during heat exposure. Compared to previous laboratory findings, the elevated thermal conductance values observed in our study indicate an active thermoregulatory response in *T. belangeri*, which enhances surface heat exchange to mitigate internal heat accumulation. This pattern has also been observed in the Hengduan Mountain small mammals *E. miletus* and *A. chevrieri* (Geng and Zhu, 2024), whereas arctic species like *Sorex cinereus* prioritize thermal insulation (Morrison et al., 1959). Additionally, this response is particularly significant for small mammals, which possess a high surface area-to-volume ratio that facilitates heat loss through their body surfaces (McNab, 2002). The increase in thermal conductance suggests that *T. belangeri* may adapt to global warming by improving surface heat dissipation, alleviating internal heat buildup, maintaining thermal equilibrium, and permitting a moderate rise in body temperature.

## 5 Conclusion

High-temperature acclimation enables *T*. *belangeri* to adapt to warmer environments by increasing body temperature to reduce the difference with ambient temperature, reducing RMR to decrease heat production capacity, enhancing heat conduction to improve heat dissipation capacity, and shifting the lower critical point of the TNZ. These adaptive strategies likely contribute to *T. belangeri* maintaining its internal thermal balance and improving its survival prospects in high-temperature environments. By comparing variations in the characteristics of the TNZ, it is observed that there has been no change in the UTNZ, suggesting a degree of adaptability to high temperatures. In addition, its TNZ is situated within tropical high-temperature regions, which further supports regarding tropical origins from the aspect of the TNZ.

Given its demonstrated thermal plasticity, we hypothesize that *T. belangeri* may expand its geographic range toward higher latitudes as global warming increases temperatures in temperate regions. Its stable UTNZ and efficient metabolic downregulation under heat stress would allow it to colonize areas previously too cool for sustained survival, potentially reaching beyond its current northern limit in the Hengduan Mountains. This expansion could lead to ecological competition with native temperate small mammals. *Tupaia belangeri*’s superior heat tolerance might allow it to outcompete these species in warmer microhabitats, altering community structures.

## Data Availability

The datasets presented in this study can be found in online repositories. The names of the repository/repositories and accession number(s) can be found below: Physiological analysis data to this submission can be found online at https://figshare.com/articles/dataset/Physiological_data_of_high-temperature_domestication_experiment_of_Tupaia_belangeri_xlsx/29312960?file=55359788.
